# Supplementary material for: Environmentally Relevant Levels of Antiepileptic Carbamazepine Altered Intestinal Microbial Composition and Metabolites in Amphibian Larvae
Source: Int J Mol Sci. 2024 Jun 25;25(13):6950. doi: 10.3390/ijms25136950 (PMC11241184; doi:10.3390/ijms25136950)
Supplement: Supplementary file 1 [file ijms-25-06950-s001.zip › ijms-3049364-supplementary.pdf]

Supplementary Information for:

Environmentally relevant levels of antiepileptic carbamazepine altered intestinal microbial composition and metabolites in amphibian larvae

Wei Dang, Jin-Hui Zhang, Zi-Chun Cao, Jia-Meng Yang and Hong-Liang Lu*

Key Laboratory of Hangzhou City for Ecosystem Protection and Restoration, School of Life and Environmental Sciences, Hangzhou Normal University, Hangzhou 311121, Zhejiang, China

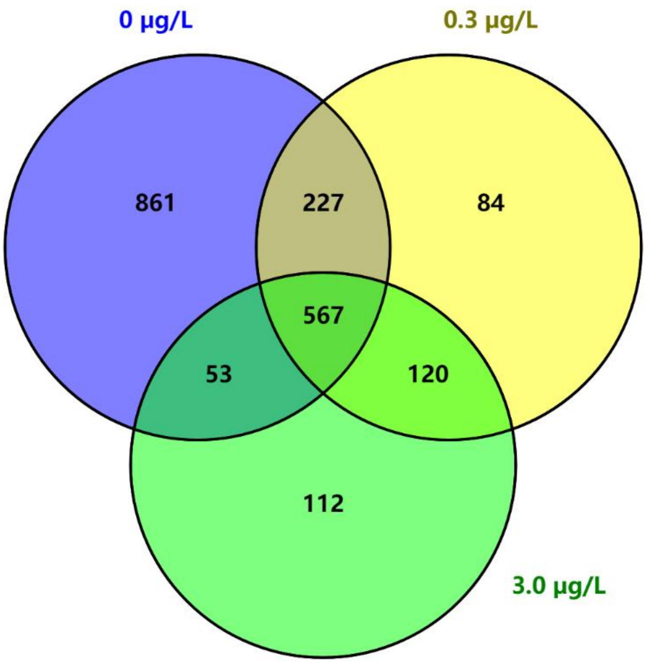

Figure S1. Obtained 16S rDNA gene sequences generated 2024 OTUs. The 0 (CTRL), 0.3 and 3.0 μg/L of carbamazepine exposure groups had 1708, 998 and 852 OTUs respectively. Among them, 567 OTUs were shared among these three groups; 861, 84 and 112 OTUs were exclusive in the 0, 0.3 and 3.0 μg/L of carbamazepine exposure groups, respectively.

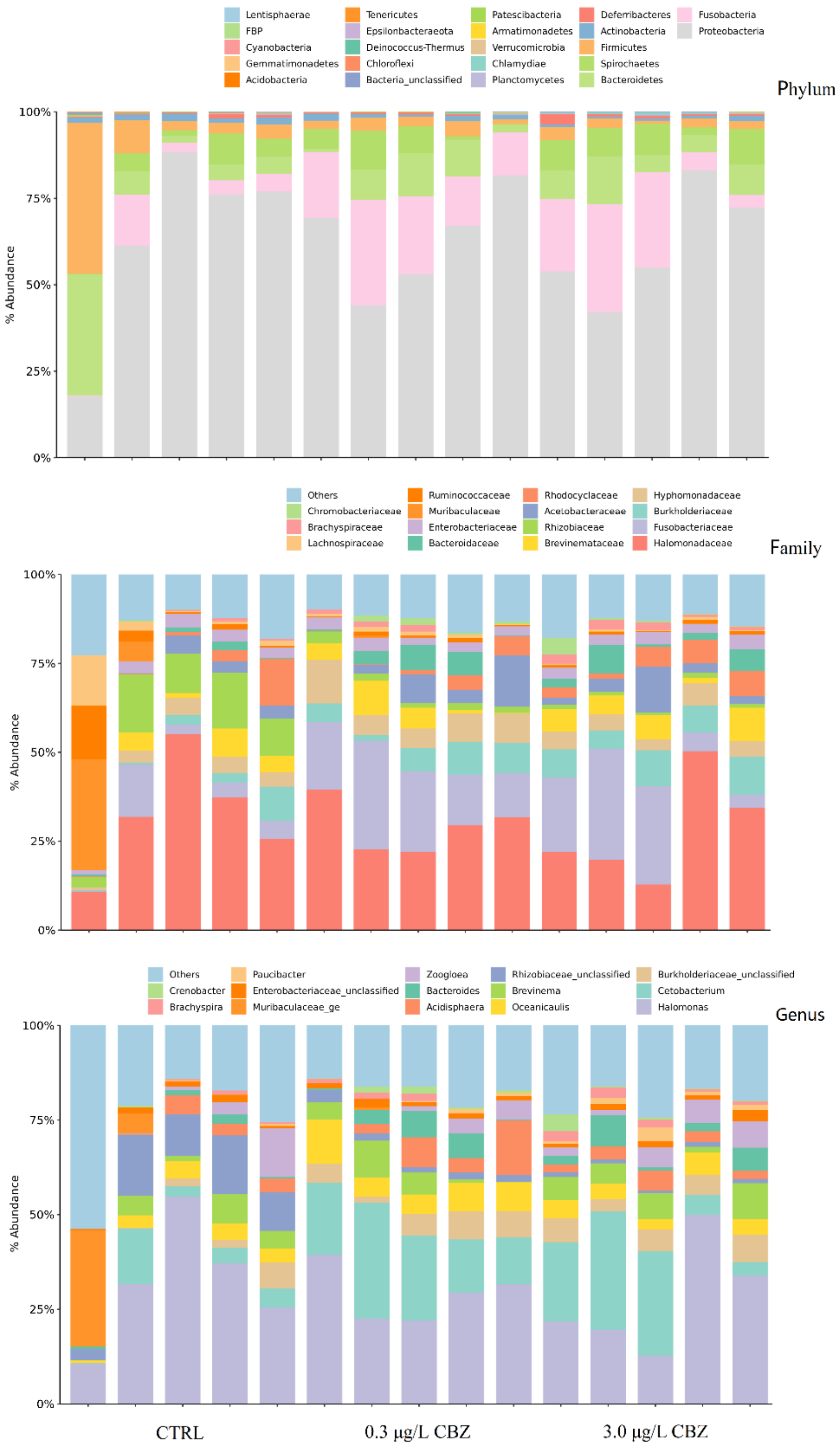

Figure S2. Relative abundances of intestinal microbiota at the phylum, family and genus levels in *Pelophylax nigromaculatus* tadpoles exposed to 0 (CTRL), 0.3, and 3.0 μg/L of carbamazepine.

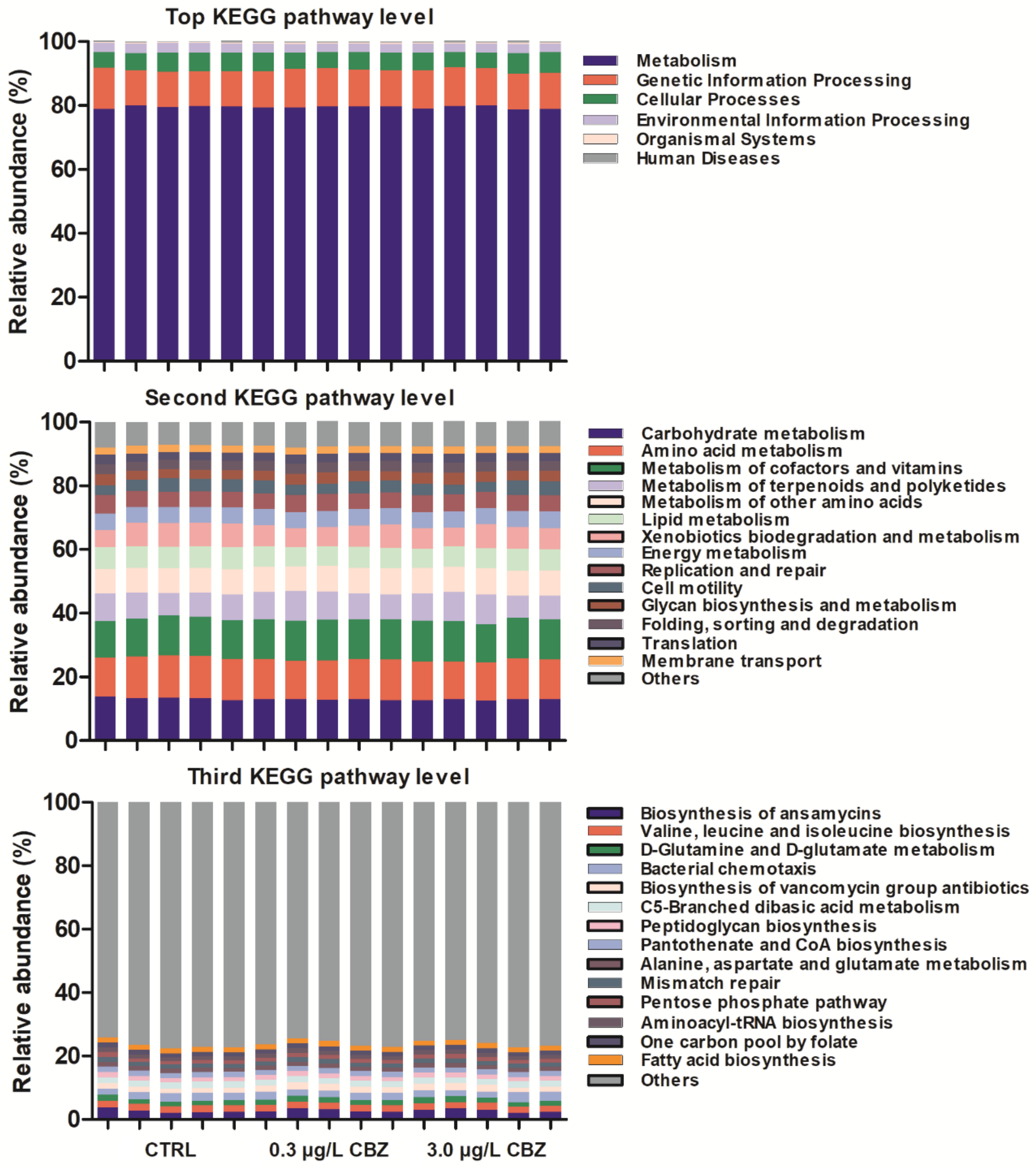

Figure S3. Relative abundance of KEGG pathways at different hierarchical levels.

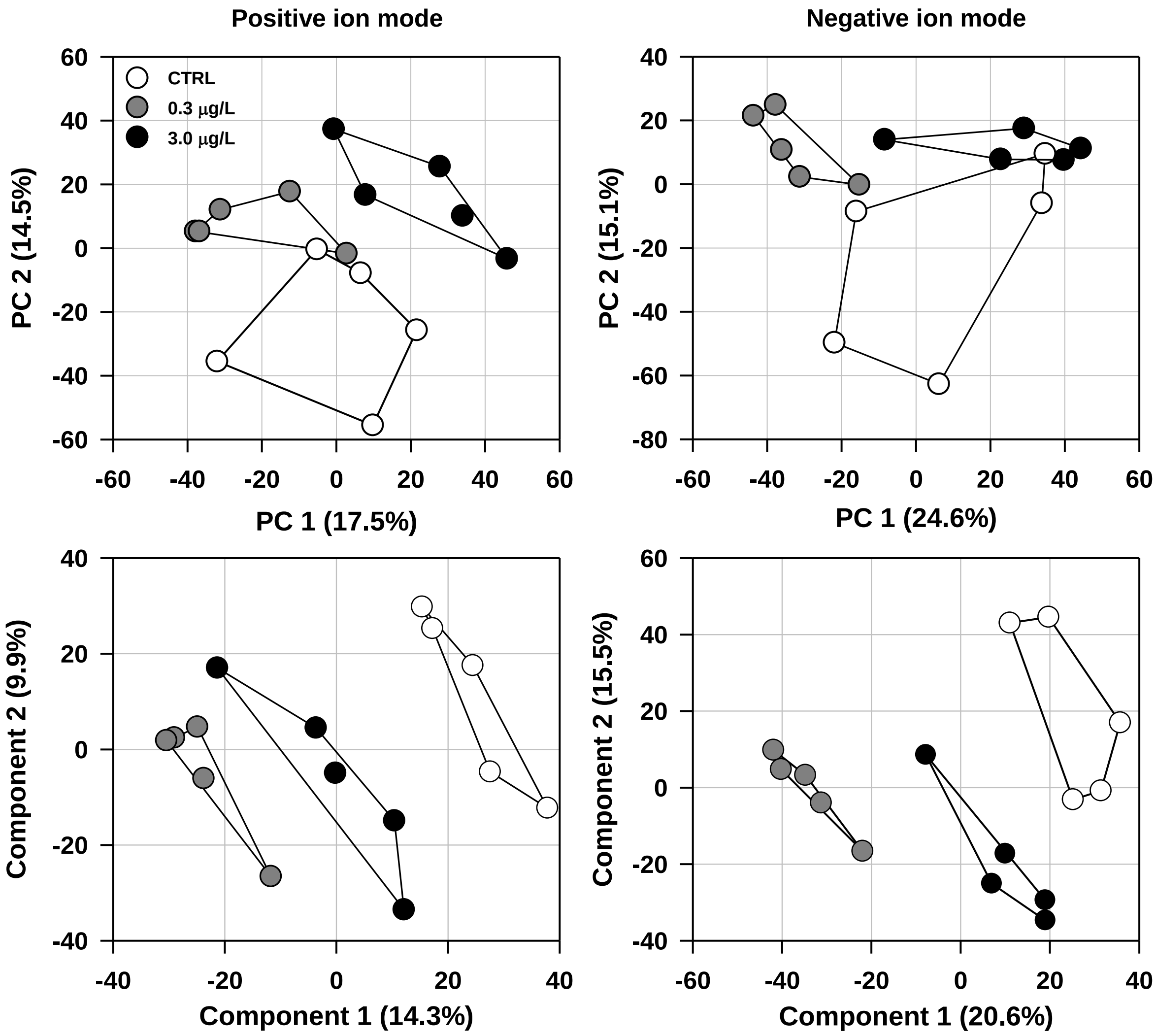

Figure S4. Score plots for principal component analysis (PCA) and partial least squares discriminant analysis (PLS-DA) analysis on intestinal metabolomic data.
